# Spatial Control of DNA Reaction Networks by DNA Sequence

**DOI:** 10.3390/molecules171113390

**Published:** 2012-11-09

**Authors:** Peter B. Allen, Xi Chen, Andrew D. Ellington

**Affiliations:** Institute of Cell and Molecular Biology, University of Texas at Austin, 1 University Station A4800, Austin, TX 78712-0159, USA

**Keywords:** reaction-diffusion, electrophoresis, chemical reaction networks, DNA circuits, strand displacement reactions

## Abstract

We have developed a set of DNA circuits that execute during gel electrophoresis to yield immobile, fluorescent features in the gel. The parallel execution of orthogonal circuits led to the simultaneous production of different fluorescent lines at different positions in the gel. The positions of the lines could be rationally manipulated by changing the mobilities of the reactants. The ability to program at the nanoscale so as to produce patterns at the macroscale is a step towards programmable, synthetic chemical systems for generating defined spatiotemporal patterns.

## 1. Introduction

Chemical reaction-diffusion networks can produce complex physical (spatial and temporal) patterns, such as those that occur during organismal development [1]. However, generalized pattern generation via designed reaction-diffusion networks is not currently feasible, in large measure because of the idiosyncrasies of chemical reactions. Specific nanometer structures have been assembled based on protein or nucleic acid folding [2], but these structures are not scalable to cellular or larger length scales. Programmed assembly of larger aggregates, such as gold nanoparticles, can produce longer-range order, but only yield highly repetitive or amorphous patterns [3].

Nucleic acid circuits have been proposed as one possible way to more generally implement chemical reaction-diffusion networks, since the strengths of interactions and the orthogonalities of reactions can be readily controlled by controlling base-pairing [4,5]. The DNA-based reactions can be treated as modular, programmable building blocks whose execution can potentially stack to form larger, more complex patterns. As a step towards the more generalized design of pattern-forming reaction networks we have examined the behavior of diffusable DNA circuits in gels. In many ways, our experimental design is analogous to the Ouchterlony double-diffusion experiment [6]. Depending on the mobility of the diffusing protein molecules, a line of immunoprecipitants occurs at a particular location in a gel. Rather than using this phenomenon to analyze interactions, we instead attempted to control reactivity and diffusion and thereby specify the locations of features. However, because diffusion is very slow at the millimeter scale we have used electrophoresis to increase the rate of feature formation from many hours to minutes. Ultimately, we were able to produce multiple static, fluorescent bands at controlled positions in space and time within the gel, and could control the location of these features by controlling the electrophoretic mobility of the underlying DNA substrates. This work is one of the first experimental demonstrations of sequence determined, specific pattern formation from bottom-up principles. 

## 2. Results and Discussion

### 2.1. Control of DNA Electrophoretic Mobility in a DNA-Functionalized Polyacrylamide Gel

Our goal was to show that we could use sequence-specific hybridization to control the migration of different DNA species in a polyacrylamide-DNA gel matrix. We first performed native polyacrylamide gel electrophoresis (native-PAGE) of a given DNA molecule with and without an antisense acridyte-DNA oligonucleotide co-polymerized in the gel. Acridyte are commercially available phosphoramidite reagents that contain allylic groups. After chemical synthesis, DNA oligonucleotides containing acridyte can be co-incorporated into acrylamide-based polymers during radical-initiated gelation [7]. DNA-acrylamide copolymers have previously been used to permanently capture targeted single-stranded DNA in gels [8], but have not been used to control relative mobility. 

**Figure 1:**
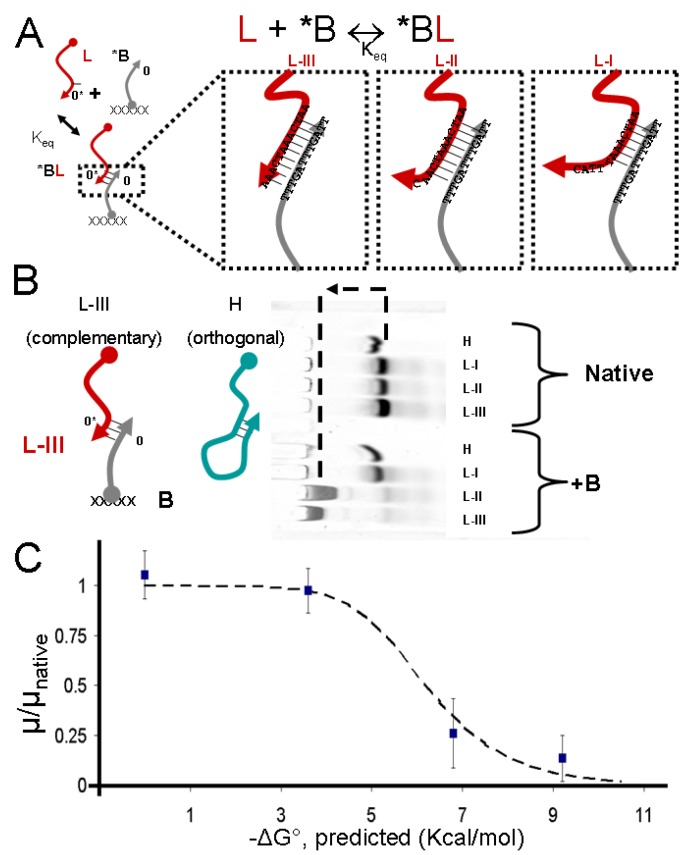
Mobility is controlled by complementarity to an immobile oligonucleotide. (**A**) The DNA acridyte *B is polymerized into the gel such that a linear mobile species L reaches local equilibration concentration between bound and unbound states. The equilibration depends on the degree of complementarity; (**B**) There is a distinct difference between the linear L species in the DNA-decorated gel and the native gel, consistent with the theoretical calculations of mobility shift due to interaction with an immobile species; (**C**) The experimental values of the mobility in the DNA-decorated gel (as a fraction of the native mobility) are plotted as a function of the theoretically calculated interaction with species B; error bars represent the width of the band. The theoretical electrophoretic mobility shift is shown as a dashed line.

A fluorescein-modified DNA oligonucleotide (**L_I_**, **L_II_**, **L_III_** or **H)** was applied to the top of native gels with and without co-polymerized DNA strands (acridyte-modified strand, **B**). Four different mobile species were assayed: three linear strands of the same overall length (38 bases), but with different sequences (**L_I_**, **L_II_**, and **L_III_** with 8, 11, and 12 bases of complementarity to **B**, respectively), and a hairpin (**H**). During this simple test of mobility, L and H are kept separate. In the absence of a co-polymerized complement, all three **L** species should have a similar characteristic mobility (µ = µ_0_) as they are negatively charged, have similar charge/mass ratios, and should present a roughly open conformation to the gel. However, in the presence of the immobile complement, **B**, each **L** species will spend some fraction of time fixed to the gel, depending on the strength of interaction. In the fixed state their mobility will be zero (µ = 0). Thus, overall mobility can be represented as a time-average between bound and unbound states (Figure 1A).

The highly complementary mobile strand (e.g., **L_III_** with 12 bases of complementarity) should move more slowly, less complementary strands (e.g., **L_I_**) would should be less retarded, and H should show no change in mobility when B is added to the gel. These predictions were borne out by the experimental results shown in Figure 1B. In the absence of an immobilized complement the three equally-sized linear strands (**L_I_**, **L_II_**, and **L_III_**) did indeed move at the same speed and migrated an equal distance during electrophoresis. In the gel with co-polymerized DNA, the three species moved at different rates and thus appeared at three different locations. Figure 1B shows the run as a single gel poured in two stages; the first stage was limited to half of the gel with a Teflon spacer. A more detailed description is available in the experimental section. The dashed arrow in Figure 1B indicates the shift in mobility from native to decorated gel. The extent of mobility change depended on their complementarity to the immobile strand (Figure 1C). The hairpin H migrated the same distance in both gels; its mobility was faster than that of the unstructured **C** strands, as expected. The dashed arrow in Figure 1B indicates the shift in mobility from native to decorated gel.

The relationship between the predicted DNA interaction strength (at the particular concentration of the species in the gel) and electrophoretic mobility is shown in Figure 1C. We calculated the free energy of interaction between each mobile species and **B** based on well-established sequence-hybridization thermodynamics [9]. We included a uniform correction to binding energies of 1.42 kCal/mol to account as an estimate of the average [1.0 kCal/mol (AA) to 2.2 kCal per mol (GC) [10]] energetic value for interactions of 1–2 bases between any of the mobile species and **B**. From the predicted equilibrium constant at room temperature and the approximate concentrations of species (5 µM for **L** and **B**) we calculated the fraction of **C** in the bound state and thereby estimated the degree to which the DNA was slowed (Figure 1C). The actual migration velocities agree well with the predictions. 

### 2.2. Design and Assay of DNA Circuits

The design of gel-interactive DNA reactions was inspired by the DNA computation field. Much recent progress in this field has entailed the use of the toehold-mediated strand-displacement reactions [11]. A strand displacement reaction occurs when a partially single-stranded DNA binds to a single-stranded DNA using a short (6–8 base-pair) “toehold” region of complementarity. This weak binding initiates branch migration, resulting in displacement of the more weakly bound strand. Complete displacement of the shorter complementary strand in favor of the longer, complete hybridized duplex is essentially kinetically irreversible, as there is no toehold to mediate reverse displacement. 

**Figure 2:**
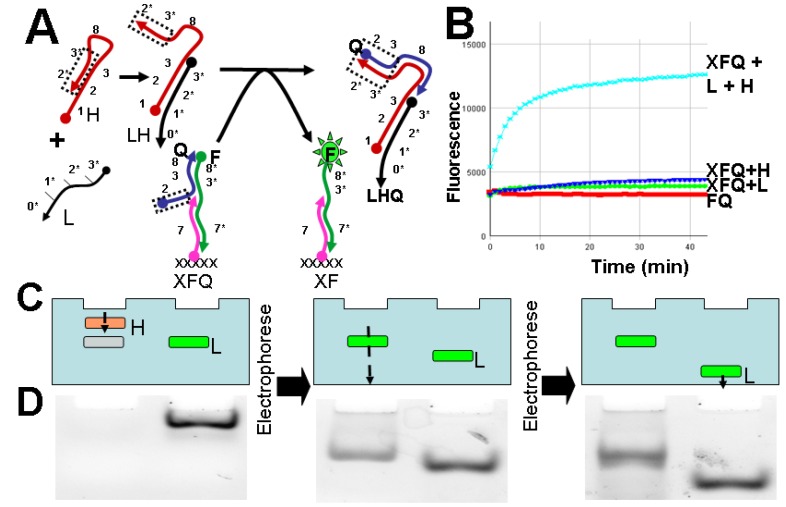
Strand displacement reactions produce a change in fluorescence. (**A**) Schematic of the design of the fluorogenic reaction. L and H interact via toehold domain 1 and complementary domain 1* to cause toehold opening. This activated L-H complex then reacts via toehold domain 2 on Q and 2* on H to abstract strand Q from FQX, leaving behind the newly fluorescent complex, FX; (**B**) Fluorescence detection of this reaction is shown over time. When all species are present (F-Q + L + H), fluorescence increases, while if any species are missing, fluorescence remains low; (**C**) A schematic diagram of the (**D**) experiment showing how L is loaded and allowed to electrophorese and is chased with H (first panel). When H reaches L, the fluorogenic reaction takes place with the XQF embedded homogeneously in the gel, generating a fluorescent feature (second panel) which stays fixed while the mobile L continues to move (third panel).

By coupling strand-displacement reactions with gel electrophoresis it should prove possible to execute computations in space, as well as time. We first present a design (Figure 2) for a fluorogenic strand displacement reaction that will produce a fixed, fluorescent feature. Using toehold-mediated strand-displacement reactions, a fluorescently-labeled DNA is displaced and thereby de-quenched only when two input strands are present (Figure 2A). In greater detail, a hairpin H and a single-stranded DNA L interact and undergo a conformational change to reveal a long, single-stranded region. This single-stranded region can in turn interact with the quencher strand Q in the fluorogenic substrate, XQF. Strand Q contains a toehold such that upon interaction with H a strand displacement reaction occurs, resulting in the fluorophore strand (F) being dequenched while remaining bound to the fixed strand (X).

Prior to performing pattern generation within a polyacrylamide gel, we confirmed that fluorogenesis occurred on the appropriate timescale in a microwell plate. The fluorescence change over time (40 min) is shown in Figure 2B. The solution-phase experiment was prepared exactly as a gel, but as a small quantity in a 384-well plate and without acrylamide prepolymer. The fluorescence was then measured with a plate reader over 1 hour. This is discussed in detail in the experimental section. When all three species were present, the fluorescence increased. Without any one of the input species, the fluorescence remained low.

### 2.3. Execution of Circuits within an Electrophoretic Gel

In order to execute the designed circuits in a spatially constrained setting, the fluorogenic reaction (Figure 2A) was executed within a gel during electrophoresis. Complex XQF was distributed homogeneously throughout the gel. It can be considered an excitable medium which then becomes fluorescent only in the presence of the localized reaction between cognate L and H species. Complex XQF was constructed in two steps, first annealing F and Q followed by adding X. This mixture was then co-polymerized into the gel. The mobile components were loaded separately with a delay between loading the reagents with slower mobilities (L species) and reagents with faster mobilities (H). The L species were allowed to migrate across almost half of the available space before loading the H species (as shown in schematic Figure 2C). As the H reagents caught up to their corresponding L reagents, the fluorogenic reaction occurred in the gel, dequenching the immobilized fluorescent oligonucleotide. The particular sequences used for this experiment were L_IV_ and H_IV_ as described in the materials and methods section below. 

The fluorogenic line generated within the gel is shown in Figure 2D. The fluorescent label makes the fluorescent species migrate slightly differently from the non-fluorescent version. The static band is not precisely level with the moving band. After a subsequent 30 min of electrophoresis, the moving band continues to migrate relative to the fluorogenic, fixed band. This single-line system is a simple version of the three line system described in further detail below. The same reaction strategy is implemented several times with different sequences. The instance is noted by a Roman numeral subscript. Figure 2 shows the general strategy (where L refers to all L species L_I_ to L_VI_; the data in Figure 2D were collected using sequence L_IV_).

### 2.4. Generation of Three Lines from Three, Orthogonal Reactions

Given the remarkable programmability of DNA circuits, we further attempted to demonstrate that through careful design multiple line generator reactions could be carried out in parallel. Three instances of the fluorogenic line generator reaction (Figure 2A) were designed with different toehold sequences (domain 1 and 1* in Figure 2A) that should have prevented cross-reaction. In addition, the sequences were designed so that the circuits would have different strengths of association with the DNA-decorated acrylamide gel, which should in turn lead to differential mobilities. 

**Figure 3:**
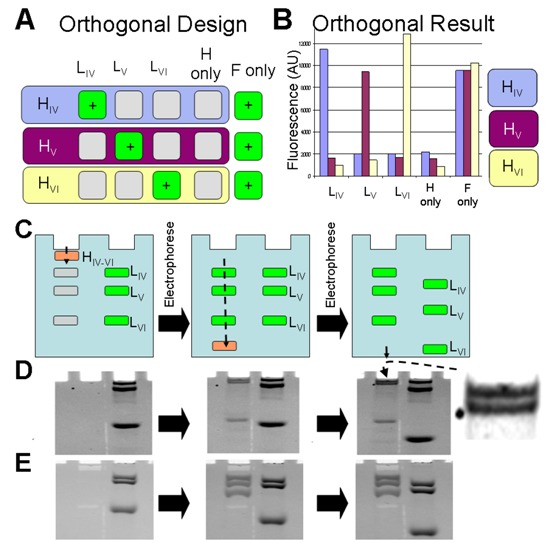
Multiple line generator reactions. (**A**) Schematic of the intended interaction network among three different instances of the fluorogenic reaction; (**B**) Experimental results showing the fluorescence increase resulting from all possible combinations of the reagents of the three reactions. Only the intended combinations show fluorescence; (**C**) The order of operations is shown as a schematic illustration; (**D**) Fluorescence images of the experimental results showing the location of the fluorescein-labeled L_IV_ L_Va_, and L_VI_ as well as the newly generated fluorogenic features in the left lane; (**E**) The same experimental protocol was applied with a different version of L_V_, L_Vb_, so that the middle, generated line (produced by L_Vb_/H_V_) is directly between those produced by L_IV_/H_IV_ and L_VI_/H_VI_.

The designed circuits were in fact largely orthogonal. The grid of possible interactions is shown in Figure 3A with “+” symbols indicating the reaction conditions that are designed to produce fluorescence. All nine reaction combinations were assayed, as well as control (H only and F only) reactions. The results are shown in Figure 3B. The cognate reactions produced fluorescence comparable to that exhibited by an equal concentration of the fluorescent strand alone (F only); non-cognate background was comparable to that with H alone (no activating C strand). 

Line formation within a gel was carried out as described above. The L species were again allowed to be loaded into the gel first and electrophoresed first, and corresponding H species were then electrophoresed after a delay. The degree of hybridization between B and the input strands L_IV-VI_ determined their relative mobilities. The intended line formation results are depicted in Figure 3C. In practice, fluorescent versions of the L_IV-VI_ species were loaded alongside the strand-exchange reactions in order to show the relative positions of the otherwise invisible L_IV-VI_ species in the reactions. The mixture of H_IV-VI_ species was applied to the top of the gel, and as each H caught up to its specific L band (Figure 3C, Panel 2) fluorescent bands appeared in the left lane in line with the solution-phase specificities (Figure 3B). The three bands were generated from the inputs L_IV_, L_Va_ and L_VI_, respectively (Figure 3D). Three lines in the gel were developed by the three reactions established as orthogonal by data presented in Figure 3B. These data were collected in solution on a plate reader (in the same manner as the kinetic data shown in Figure 2B). These same orthogonal reagents were used in the gel and cross reactivity is thus assumed to be minimal. The lines produced by L_IV_ and L_Va_ were very close together (see Figure 3D inset). In order to show that changes in sequence can be used to rationally alter the relative positions of the lines, we adjusted the sequence of L_Va_ and created a new version L_Vb_ new version, L_Vb_ with lower energy of interaction with immobilized oligonucleotide B. This simple change altered the relative position of the middle line (produced by L_Vb_ and H_V_) such that it was now produced at a distance halfway between the other two (see Figure 3E). The fluorescent features produced by the designed circuits held fast in the gel, while in contrast free, fluorescent species continued to migrate (compare with right lane, Figure 3C, Panel 3). Because the mobility of L_V_ is different, the ideal migration distance for visualization was also different. We electrophoresed for a different amount of time between the frames shown in Figure 3D and Figure 3E. The time spacing was chosen arbitrarily for presentation clarity.

### 2.5. Modular Manipulation of DNA Circuits; Fixing a Mobile, Fluorescent Species

**Figure 4:**
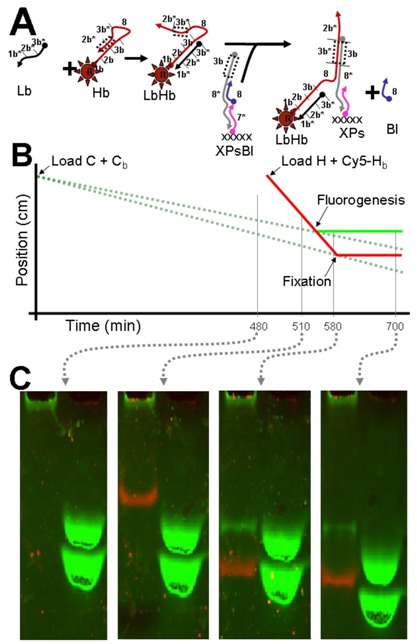
DNA reactions cause fluorescence and mobility changes* in situ*. (**A**) Schematic illustration of the fixation reaction that leads to a decrease in the mobility of Cy5-labeled Hb; (**B**) Graph showing the motion of the DNA species over time in the gel. The L species are injected and electrophorese down the gel. H species are then injected; they move much faster. The lines intersect and reactions occur at specific locations; (**C**) Two-color fluorescence images of the experimental results showing the location of the fluorescein-labeled L (green) and Cy5-labeled Hb (red) species as they migrate. As above, fluorescein-labeled L in the right lane indicate the locations of the invisible L species. As H overtakes L, the fluorogenic reaction (above) de-quenches embedded fluorescein and creates a new green line. Hb, labeled with Cy5 (red) can be seen to arrest its motion after frame 3.

The modularity of DNA circuits allows the different components to be reorganized in gel-based reactions. Previously, reactions were designed to de-quench a fluorescent tag at a specific locale in the gel; we next attempted to show that a different circuit could be readily designed that would lead to fixation of a mobile, fluorescent species at a specific locale. We simultaneously ran the green, fluorogenic reaction above (presented in Figure 3) with a second, orthogonal reaction visualized with red fluorescence. This reaction shows that a moving entity (red fluorescent, Cy5 labeled hairpin, Cy5-Hb) can react and become immobile. In the designed circuit, the fixation reaction (Figure 4A) occurs when Hb is opened upon meeting an input molecule, Lb, within the gel. Together they form a new complex, Lb-Hb that can displace an immobilized blocker strand (Bl, covering domain 8* on Ps) to create a 25-base-pair duplex.

The fixation reaction is orthogonal to the previously described fluorogenic line generator, and the two reactions can be executed in the same gel at the same time. To demonstrate this, L_IV_ and Lb (slow species) were loaded into the gel and after a delay H and Cy5-Hb were loaded behind them. The relative mobilities of these different species are represented in the lines shown in Figure 4B. This graph is a semi-quantitative illustration based on the approximate (+/− 5 min) time recordings of the fluorescence images. The intersection of the dashed (input) and solid (transducer) lines indicate where immobile, fluorogenic lines should occur, either due to uncovering of immobilized fluorophores or to fixation of Cy5-Hb.

Experimental results for this comparison of circuits are shown in Figure 4C. As expected, the red Cy5-H is apparent throughout the run as it moves past the green-fluorescent band uncovered by the L_IV_/H_IV_ reaction (compare panels 2 and 3), and then stops when it reacts with Lb and encounters an immobilized antisense strand. The immobilization of the fluorescent bands in the left lanes is apparent when compared with the mobile, green fluorescent species in the right lanes. After fixation, Cy5-Hb moves only a very small distance during the final 100 or so minutes of electrophoresis (compare panels 3 and 4) due to hairpin-mediated dissociation. The fixed Hb is unlike the fluorogenic reaction in which a linear, fixed fluorophore is de-quenched. Hb has a hairpin conformation as well as a linear, bound, fixed conformation. As some fraction of Hb assumes the hairpin conformation, it can decrease its hybridization to the fixed strand and increase its effective mobility.

## 3. Experimental

### 3.1. Control of DNA Electrophoretic Mobility in a DNA-Functionalized Polyacrylamide Gel

Native acrylamide gel (5% acrylamide prepolymer, BioRad, Hercules, CA, USA) was prepared in running buffer (44 mM Tris base, 44 mM boric acid, 1 mM disodium EDTA, 10 mM magnesium acetate, 5% w/v glycerol (all from Sigma-Aldrich, St. Louis, MO, USA). Acrylamide prepolymer (2.5 mL) was treated with 2.5 µL of TEMED and 25 µL of APS (Sigma-Aldrich) to initiate radical polymerization. This was rapidly mixed with the DNA to be immobilized (e.g., B at 5 µM) and allowed to polymerize between the glass plates in the gel rig. The concentration of the immobilized DNA was chosen to be 5 µM because the concentration of B must be comparable to the concentration of the mobile DNA; mobile species concentrations were chosen in the micromolar range in order to achieve a reasonable reaction rate. A Teflon block was inserted into the rig during polymerization so that only half of the rig was filled with polymer. Once the gel polymerized, the Teflon block was removed. A second 2.5 mL of native gel was prepared equivalently but without DNA and treated with TEMED and APS. This native gel was then introduced to the empty volume and allowed to polymerize.

We used this two-part gel to show that the electrophoretic mobility depends on the complementarity to the gel-immobilized DNA. The two-part gel (native and DNA-acrylamide-co-polymer) was loaded with fluorescein-modified DNA samples. Fluorescein-modified DNA was used because the co-polymerized DNA makes post-electrophoresis staining ineffective. A set of 4 samples (40 pmol each, L_I_, L_II_, L_III_, and H) was loaded into the DNA-acrylamide (one sample per well) and a second identical set of samples was loaded into the native acrylamide side. The gel was subjected to an electric field at 85 v for 2 h and then were scanned with a Storm scanner (GE Healthcare, Little Chalfont, UK). 

To compare these results to theory, we acquired the migration distances from the top of the well using ImageJ (NIH). The migration distances of the bands in the DNA-acrylamide co-polymer gel are shown as a fraction of the migration distances in the native gel. We used a line-intensity scan to measure the half-width-half-max of the bands, which are presented as relative error bars in Figure 1C.

We computed the standard interaction energy with OligoCalc [9] using the sequence of the L species extension and the partially complementary sequence of the immobilized X species. The energy (plus a constant energy of 1.6 kCal/mol of interaction to account for the conserved regions of L) was used to calculate the dissociation constant (K_d_) with the Gibbs free energy identity. The equilibrium constant, temperature, and initial experimental concentrations of mobile and immobile DNA ([L]_0_ and [B]_0_, respectively, taken to be the experimental values of 5 µM for each) were used to analytically solve for a local equilibrium concentration of L and B. This was then used to calculate the fraction in the bound and unbound state. The unbound fraction (F_unbound_ = 1 − [BL]/[L]_0_) was plotted in Figure 1C as the dotted line:

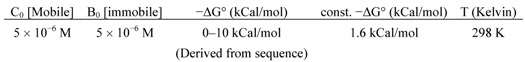


### 3.2. Solution Assay of Fluorogenic DNA Circuits

DNA complexes were prepared with oligonucleotides from Integrated DNA Technologies (IDT, Coralville, IA, USA) as prepared by the manufacturer, with the exception of hairpin (H species) and quencher DNA (Q), which were gel purified and ethanol precipitated. To prepare complex XQF, one volume of 40 µL BND buffer (137 mM sodium chloride, 2.7 mM potassium chloride, 12 mM sodium phosphate and 10 mM added magnesium chloride at a pH of 7.4) was prepared containing 10 nM F and a slight excess (11 nM) of Q. This solution was annealed by heating to 80 °C for 3 min and then cooled at a rate of 0.1 °C per second. A further excess (12 nM) of X was then added and mixed by gently vortexing. This solution was then split into four wells in a 384-well plate. 3 µL of BND containing 33 nM of the appropriate version of H, both H and L, or buffer alone were then added to the FQX complex. Time-course fluorescence measurements with excitation at 490 nm and emission at 510 nm were then acquired at 1 min intervals over approximately 60 min using a TECAN SAFIRE plate reader (TECAN, Männedorf, Switzerland). 

### 3.3. Execution of Circuits within an Electrophoretic Gel

DNA complexes were prepared with oligonucleotides from IDT as prepared by the manufacturer, with the exception of hairpin (H species), fluorescein (F) and quencher DNA (Q), which were gel purified and ethanol precipitated. XQF complex was prepared as above, and then the gel rig was prepared with the XQF complex (100 nM), and B (5 µM) polymerized into the gel as above. Fluorescein-modified and native versions of L_V_ were added to adjacent wells (10 pmol each) and electrophoresed at 75 v for 30 min. A 10 pmol quantity of H_Va_ was then added to chase the non-fluorescent version of L. Electrophoresis continued at 75 v for 60 min, with a fluorescence image acquired every 10 min using a FluorChemQ imaging station (ProteinSimple, San Jose, CA, USA).

### 3.4. Generation of Three Lines from Three, Orthogonal Reactions

XQF complex was prepared as above, and then the gel rig was prepared with the XQF complex (100 nM) and B (5 µm) polymerized into the gel as above. Fluorescein-modified and native versions of L_IV_, L_V_, and L_VI_ were added to adjacent wells and electrophoresed at 80 v for 80 min. A mixture of H_IV_, H_Va_, and H_VI_ were then added to chase the non-fluorescent versions of L. Electrophoresis continued at 80 v for 200 min with a fluorescence scan acquired every 15–20 min using a Storm scanner (GE Healthcare).

### 3.5. Modular Manipulation of DNA Circuits; Fixing a Mobile, Fluorescent Species

XQF complex was prepared as above, and XPsBl was prepared equivalently. Then the gel rig was prepared with the XQF complex (100 nM), XPsBl (100 nM), and B (5 µm) polymerized into the gel as above. Fluorescein-modified and native versions of L_IV_, L_Vb_ were added to adjacent wells and electrophoresed at 80 v for 80 min. A mixture of H_IV_, and Cy5-Hb was then added to chase the non-fluorescent versions of L. Electrophoresis continued at 80 v for 200 min, with a fluorescence image acquired every 15–20 min using a 2-color gel imaging station FluorChemQ (ProteinSimple).

### 3.6. DNA Sequences

Oligonucleotides were acquired from IDT and purified by denaturing gel electrophoresis as described above:

B 5'-acrydite-TTTGATTTGATT
L_I_ 5'-fluorescein-CGACATCTAACCTAGCAAATACGATGAATCAAATTTAC
L_II_ 5'-fluorescein-CGACATCTAACCTAGCCTCCTCCTCCAATCAAATCAAC
L_III_ 5'-fluorescein-CGACATCTAACCTAGCCTCCTCCTCCAATCAAATCAAA
H 5'-GTCAGTGATGCTAGGTTAGATGTCGCCATGTGTAGACGACATCTAACCTAGCCCTTGTCATAGA GCAC
L 5'-CGACATCTAACCTAGCATCACTGACAATCAAATCTTC
F 5'-GAATTGTCAGACTTCTCCTTGTCATAGAGCAC-3’-fluorescein
X 5'-acrydite-AGAAGTCTGACAATTC
Q 5'-Black Hole Quencher-GTGCTCTATGACAAGGGCTAGGTT
Ps 5'-GTTTACCG CTCCCTCCTTTTAGTTGGAATTGTCAGACTTCT
Bl 5'-CAACTAAAAGGAGGGAG
Hb 5'-Cy5-TCGTATTTGGTTTTTGGTTTACCGCAACTAAAAGGAGGGAGCGGTAAACCAAAAACC
Lb 5'-CGGTAAACCAAAAACCAAATACGAAATCAAATCAAA
H_VI_ 5'-GGAGGAGGAGGCTAGGTTAGATGTCGCCATGTGTAGACGACATCTAACCTAGCCCTTGTCATA GAGCAC
L_VI_ 5'-fluorescein-CGACATCTAACCTAGCCTCCTCCTCCAATCAAATCAAC
H_V_ 5'-CATCGTATTTGCTAGGTTAGATGTCGCCATGTGTAGACGACATCTAACCTAGCCCTTGTCATAG AGCAC
L_Va_ 5'-fluorescein-CGACATCTAACCTAGCAAATACGATGAATCAAATCATG
L_Vb_ 5'-fluorescein-CGACATCTAACCTAGCAAATACGATGTCCCAAATCAAA
H_IV_ 5'-CACCACCCACGCTAGGTTAGATGTCGCCATGTGTAGACGACATCTAACCTAGCCCTTGTCATA GAGCAC
L_IV_ 5'-fluorescein-CGACATCTAACCTAGCGTGGGTGGTGAATCAAATCTTC

## 4. Conclusions

We have developed a modular set of DNA circuits that can execute during gel electrophoresis to produce static, fluorescent macroscale patterns. The DNA circuits executed orthogonally to yield differing features in space and time, and the positions of the features could be controlled by programming the diffusivity of the underlying nucleic acid components. The robustness of the method allowed for entirely different DNA circuit configurations to be successfully explored, suggesting that this approach may allow more complex reaction-diffusion networks to first be ‘breadboarded’ in gels. Most importantly, for one of the first times outside of biology or crystallography, a connection between biomolecular programs and macroscale features was achieved.

Ultimately, nucleic acid circuits executing in reaction-diffusion networks might be the basis of designed Turing-like systems [12] that would produce patterns of arbitrary complexity through manipulation of underlying molecular properties and algorithms. As greater control over reaction-diffusion networks and pattern formation is obtained, objects that self-assemble at the nanoscale, such as DNA origami [2], might become building blocks in larger assemblies. Patterns self-assembled in gels might prove to be the basis for the formation of materials, since selective cross-linking within gels by self-assembled DNA helices [13] could stabilize some regions and allow others to be washed away, the inverse of many 3D extrusion strategies for prototyping materials and shapes [14].

## References

[1] Kondo S., Miura T. (2010). Reaction-Diffusion Model as a Framework for Understanding Biological Pattern Formation. Science.

[2] Rothemund P.W.K. (2006). Folding DNA to create nanoscale shapes and patterns. Nature.

[3] Macfarlane R.J., Lee B., Jones M.R., Harris N., Schatz G.C., Mirkin C.A. (2011). Nanoparticle Superlattice Engineering with DNA. Science.

[4] Yin P., Choi H.M.T., Calvert C.R., Pierce N.A. (2008). Programming biomolecular self-assembly pathways. Nature.

[5] Phillips A., Cardelli L. (2009). A programming language for composable DNA circuits. J. Roy. Chem. Soc..

[6] Ouchterlony O. (1958). Diffusion-in-gel methods for immunological analysis. Prog. Allergy.

[7] Rehman F.N., Audeh M., Abrams E.S., Hammond P.W., Kenney M., Boles T.C. (1999). Immobilization of acrylamide-modified oligonucleotides by co-polymerization. Nucleic Acids Res..

[8] Chan A., Krull U.J. (2006). Capillary electrophoresis for capture and concentrating of target nucleic acids by affinity gels modified to contain single-stranded nucleic acid probes. Anal. Chim. Acta.

[9] Kibbe W.A. (2007). OligoCalc: An online oligonucleotide properties calculator. Nucleic Acids Res..

[10] SantaLucia J. (1998). A unified view of polymer, dumbbell, and oligonucleotide DNA nearest-neighbor thermodynamics. Proc. Natl. Acad. Sci. USA.

[11] Zhang D.Y., Turberfield A.J., Yurke B., Winfree E. (2007). Engineering entropy-driven reactions and networks catalyzed by DNA. Science.

[12] Turing A.M. (1952). The Chemical Basis of Morphogenesis. Philos. T. Roy. Soc. B.

[13] Zhu Z., Wu C., Liu H., Zou Y., Zhang X., Kang H., Yang C.J., Tan W. (2010). An Aptamer Cross-Linked Hydrogel as a Colorimetric Platform for Visual Detection. Angew. Chem. Int. Ed. Engl..

[14] Hoque M.E., Chuan Y.L., Pashby I. (2011). Extrusion based rapid prototyping technique: An advanced platform for tissue engineering scaffold fabrication. Biopolymers.

